# A Novel Multi-Modal Teleoperation of a Humanoid Assistive Robot with Real-Time Motion Mimic

**DOI:** 10.3390/mi14020461

**Published:** 2023-02-16

**Authors:** Julio C. Cerón, Md Samiul Haque Sunny, Brahim Brahmi, Luis M. Mendez, Raouf Fareh, Helal Uddin Ahmed, Mohammad H. Rahman

**Affiliations:** 1 Mechatronics Engineering, Universidad Nacional de Colombia, Cra 45, Bogatá 111321, Colombia; 2 Computer Science, University of Wisconsin Milwaukee, Milwaukee, WI 53212, USA; 3 Electrical Engineering, College Ahuntsic, Montreal, QC 9155, Canada; 4 Electrical Engineering, University of Sharjah, University City, Sharjah 27272, United Arab Emirates; 5 Biorobotics Laboratory, Mechanical Engineering, University of Wisconsin Milwaukee, Milwaukee, WI 53212, USA

**Keywords:** assistive robot, teleoperation, kinect, meta quest, NAO robot, motion capture, humanoid robot, ROS

## Abstract

This research shows the development of a teleoperation system with an assistive robot (NAO) through a Kinect V2 sensor, a set of Meta Quest virtual reality glasses, and Nintendo Switch controllers (Joycons), with the use of the Robot Operating System (ROS) framework to implement the communication between devices. In this paper, two interchangeable operating models are proposed. An exclusive controller is used to control the robot’s movement to perform assignments that require long-distance travel. Another teleoperation protocol uses the skeleton joints information readings by the Kinect sensor, the orientation of the Meta Quest, and the button press and thumbstick movements of the Joycons to control the arm joints and head of the assistive robot, and its movement in a limited area. They give image feedback to the operator in the VR glasses in a first-person perspective and retrieve the user’s voice to be spoken by the assistive robot. Results are promising and can be used for educational and therapeutic purposes.

## 1. Introduction

Human–robot interactions have been one of the most studied fields in robotics during the last decade, having great technological developments in areas such as the teleoperation of machines, which has allowed humankind to reach places and perform tasks that were previously considered impossible in a comfortable and safe way [1,2,3]. In addition, with the introduction of assistive humanoid robots to the market, a new spectrum for teleoperation has been generated, having a great focus on this type of machine, as they can have great potential in educational or even therapeutic fields [4,5,6,7,8,9].

One of the most common assistive humanoid robots used to achieve this goal is the NAO robot, having an advanced high-level programming interface (choreography) that can be easily introduced to people with no programming skills [10]. However, when the robot must perform more human-like movements, there are two methods to do: by giving it a series of steps to accomplish the duty, which take a long time and increases its difficulty and complexity, or by using sensors to track and retrieve the human motion to be performed by the robot. This last option has more advantages; it allows the human operator to directly manipulate the robot, increasing their decision making and adaptability in any given task.

Many approaches have been made in research and developments on imitation-based systems with the NAO robot, introducing many sensing devices that can track human motion in real-time as the mXsens MVN full-body motion capturer [11] or the MoCap [12]. However, these devices are costly and not very affordable, so most of the real-time teleoperation work has been conducted using depth cameras, such as the Kinect [13,14,15] as it is very cost-effective. Nevertheless, some of these systems are stationary and with minimal focus on mobility and other teleoperation systems, which can be done with direct visual contact on the robot without considering an immersive experience for the operator.

This work proposes a teleoperation concept in an ROS environment with two different modalities of use that can be changed with a Joycon controller. One is used to teleoperate the assistive robot with the Joycon’s gamepad for long-distance tasks. The other is used for short-distance tasks, which, in addition to upper body imitation, would enable the operator to walk and turn in a specific, close area in real time. The goal is accomplished by using a Kinect sensor V2 to track the traveling movements of the operator’s arms, and the motion of both their shoulder and elbow joints to be sent to the robot. At the same time, the Joycons will control both the wrist joints and hands. Furthermore, to achieve an immersive experience, it is connected to a Meta Quest to retrieve the camera image of the robot and control its head joints and the use of the Kinect microphone to speak through the robot. The inverse kinematic used in the research is an innovative approach inspired by natural motion and can imitate natural human movement. This integrated system can be used for academic purposes. However, it could potentially be used in occupational therapy, carrying out interactive therapy sessions with children who have a disorder on the autistic spectrum. With the therapists using this system to teleoperate with the assistive robot, they will interact with the patients through NAO, which could be more comfortable for the children and improve their communication skills.

## 2. State of the Art

There have been many approaches to teleoperate the assistive robot NAO using different sensors to acquire human skeleton movement data [11,12,14,15,16,17,18,19].

Stanton, Bogdanovych, and Ratanasena present and evaluate a method for teleoperating an assistive humanoid robot via a full-body motion capture suit, called “mXsens MVN full-body motion capturer”, tracking each joint of each limb separately through motion sensors embedded in the suit in [11]. Nuñez, Dajles, and Siles, in [12], use markers displayed all over the human operator to track body motion captured by a MoCap with cameras of the series Prime 41 from OptiTrack.

However, plenty of teleoperation developments have been conducted with Kinect sensors [17], which are low-cost and easy to find in the market. Mota, Moreira, and Pereira do Nascimento [18] use the Kinect to track the upper limbs of the operator to be mapped in the NAO Robot. Li et al. [20] take a similar approach to teleoperating the assistive robot by tracking the superior limbs of the operator and solving the inverse kinematics of the robot limbs. Mukherjee, Paramkusam, and Dwivedy [19] take three different approaches to solve the arm kinematics of the operator to be used in the robot arms. The first one is a geometrical approach, the second one is by solving Inverse kinematics using adaptive neuro-fuzzy inference systems (ANFIS), and the third one is by resolving the inverse kinematics using the iterative Jacobian method. Nevertheless, all of these researches focused on the movement of the limb as just stationary. For this reason, developments have included interactive poses with the Kinect sensor to control the assistive robot’s movements in the work area. Rodriguez et al. developed a software package that allows real-time speech-based guidance and gesture-based teleoperation in [15]. Almetwally and Mallem [14] used leg and arm gestures to interact with the teleoperation system, which allow navigation of the robot in a close area.

After analyzing these developed platforms, it can be seen that plenty of the approaches have been made almost exclusively with Windows software since there is convenience in finding the device drivers in this platform. Many researchers have used Linux software to connect the sensor devices and the robot by ROS, which has the great advantage of a messaging system that uses communication between distributed nodes via the anonymous publish/subscribe mechanism resulting in better connectivity between devices [21], even though these have to be used with third-party drivers created by the Linux programmer community [22]. Avalos et al. [13] develop a method to teleoperate the NAO robot using ROS and Naoqi bridges, although using other Windows computers to handle the data acquisition of the Kinect sensor. Ajili, Mallem, and Didier in [23] use a gesture recognition system based on the Hidden Markov Model method to teleoperate NAO. Ref. [14,15] also develop their research in an ROS environment, using the free source package to interact with the robot NAO.

It is also worth noting the investigations of Sripada et al. [24], which propose a teleoperation concept with upper body imitation that would enable users to walk forward or backward, and also turn the humanoid robot with 20 DoF (Degree of Freedom) in real-time. Fritsche et al.  [25] provide a first-person teleoperation framework for humanoid robots using a system based on Kinect, Meta Quest, and SensorGlove to an iCub robot.

In this work, an approach that integrates the aforementioned study’s strengths is proposed by providing a first-person teleoperation framework for the assistive robot NAO through a real-time teleoperating system based on Kinect sensor V2, Meta Quest, and Joycon controllers in an ROS ecosystem. It allows the operator to change between two modalities to control the assistive robot’s long and short-distance tasks. Controlling the robot’s movement and orientation with the Joycons in the first modality, and controlling the arms, head, whole body position, and orientation in the other modality, where the operator can see through the robot head camera and speak via the microphone.

## 3. Preliminaries

### 3.1. Humanoid Assistive Robot

**Figure 1:**
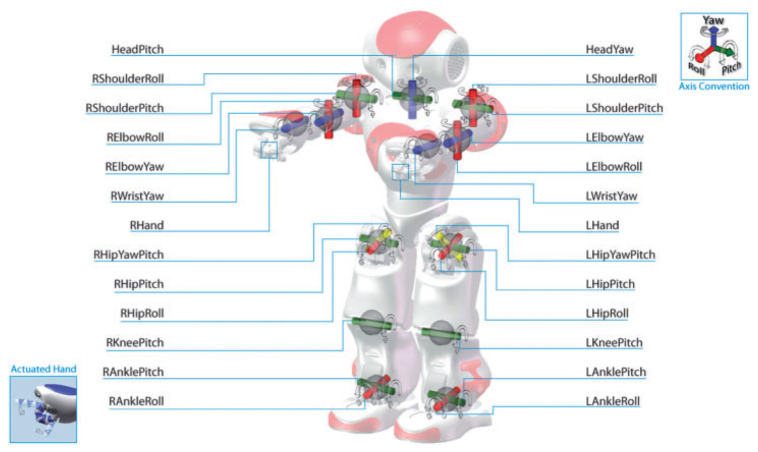
All joints in the NAO robot at initial positions [28].

**Figure 2:**
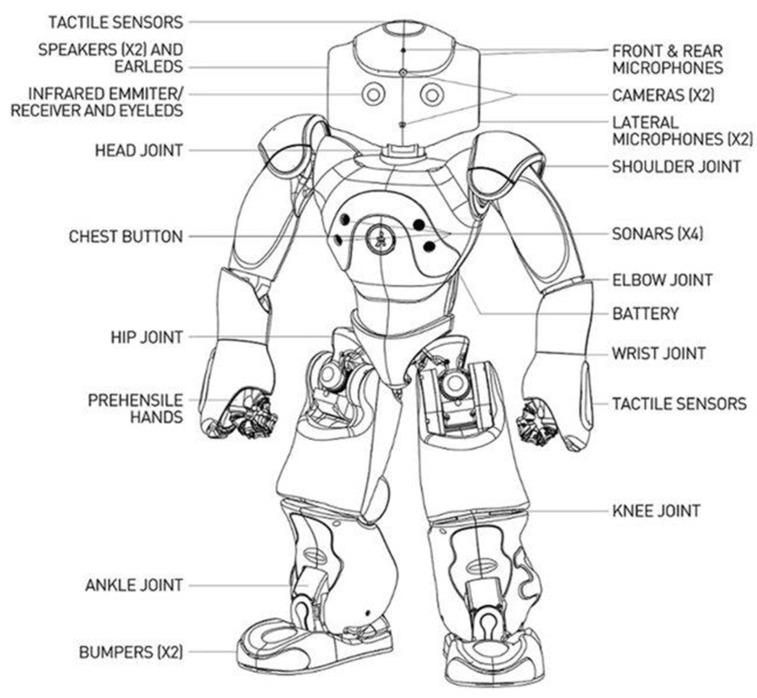
All sensors in NAO robot [29].

The NAO robot from SoftBank Robotics was chosen to achieve the teleoperation task with an assistive robot [26]. With 25 degrees of freedom (DOF), NAO is 4.5 kg in weight and 573.2 mm in height, making it the lightest and smallest robot in the Aldebaran catalog. As an educational robot, for its main purpose, NAO can perform a series of tasks from image retrieving and analysis to walking and moving toward goal points and speech recognition. This robot can be programmed by the Choreagraphe suite or with the Naoqi SDK, which is available in different programming languages, such as Python or C++ [27]. All the joints of the NAO robot are depicted in Figure 1, and all the sensors are indicated in Figure 2.

### 3.2. Robot Operating System (ROS)

**Figure 3:**
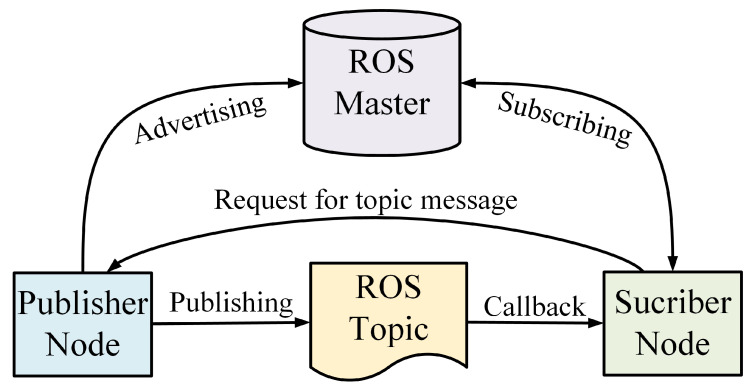
A typical ROS Model: System components.

The Robot Operating System, commonly known as ROS, is a framework for robot software development. The system is based on a modular concept. Modules are named nodes in ROS, and nodes communicate via topics, services, or actions following an asynchronous publisher/subscriber protocol. In addition, ROS gives the advantage of working in different languages, such as python or C++, and performs a reliable connection between them [30]. A typical ROS model and it’s components are illustrated in Figure 3.

### 3.3. Kinect Sensor

**Figure 4:**
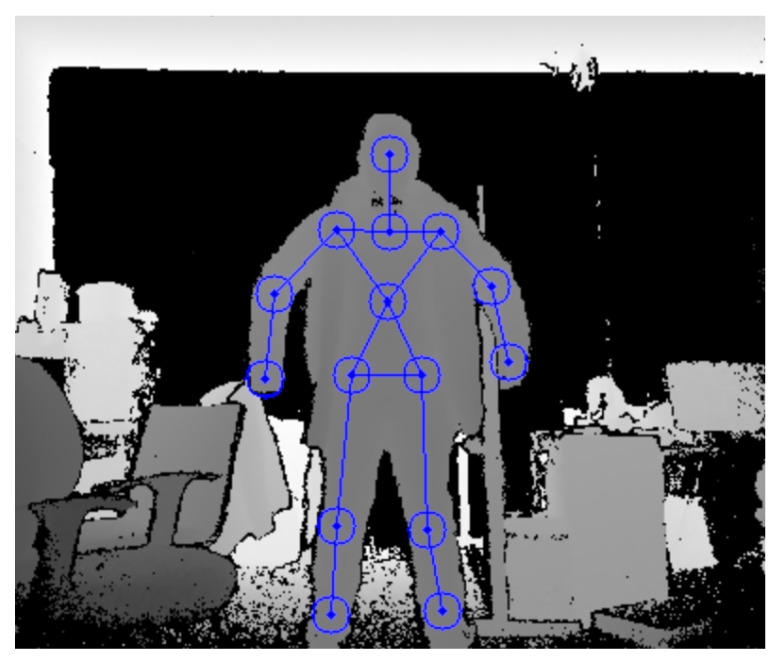
Skeleton tracking with the Kinect sensor and OpenNI2.

To retrieve motion body data of the operator, the Microsoft Kinect V2 [31] was used. It combines a camera and a depth sensor to capture depth, color, and IR images to retrieve 3D object data. Both the color and depth image are transformed into a common frame, with the origin located at the center of the sensor, called *Camera Space* [32]. These devices are commonly used for body tracking and are more affordable compared to full body tracking suites. Nonetheless, this kind of sensor has two disadvantages. First, data acquisition is noisy and has to be treated before it is used in tasks with precision requirements. Second, by relying on the sensor’s field of vision, the data acquired depends on the tracking of the body that is not occluded by other objects or even by other parts of the body. By working in a Linux environment, there is often official support with drivers of devices such as the Kinect sensor or the Meta Quest, which usually worked with just Windows platforms or their game console device. To work with the Kinect, finding a third-party driver is necessary that can retrieve depth images and allow human body recognition with this sensor. OpenNI2 (Open Natural Interaction) is an open-source software development kit for the RGB-D sensor as the Kinect sensor or the PrimeSense camera [33]. In addition, NiTE2 is a middleware, which has features such as human detection, posture estimation, hand tracking, and gesture detection. For this project, it is used to achieve the Cartesian coordinates of 15 identifiable joints [34]. Furthermore, it was used with the python bindings because it is easier to program in the ROS environment, which was coded in python in its majority. An example of human body tracking is shown in Figure 4.

### 3.4. Meta Quest

To achieve a first-person immersive teleoperation experience, a Meta Quest was used. It is a virtual reality headset created by Meta [35]. It uses a combination of three-axis gyros, accelerometers, and magnetometers, which make it capable of absolute (relative to Earth) head orientation tracking without drift. The gyroscope data are retrieved in quaternions, which are transformed into Euler angles to move the head joints. Due to the unavailability of SDK in Linux, OpenHMD drivers are implemented. They provide a Free and Open Source API for immersive technology [36]. It supports a wide range of devices, such as the Meta Quest. It is used with the python bindings by Lubosz Sarnecki to retrieve the device’s orientation and use its display in extended mode (as a second display for the computer) [36].

## 4. Control Architecture and Theoretical Analysis

**Figure 5:**
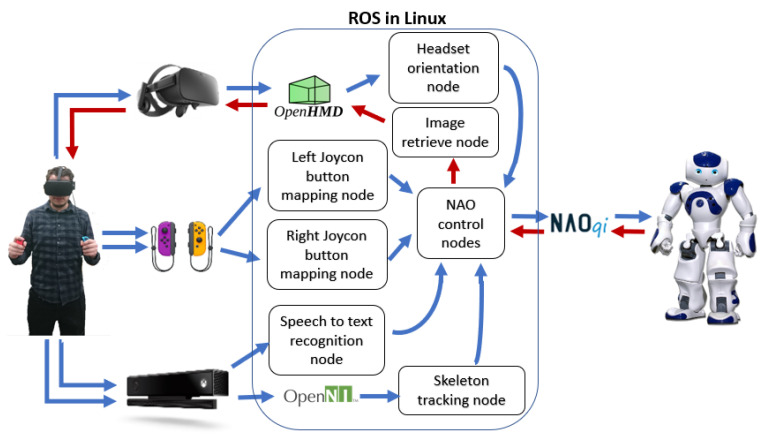
Modular software architecture of the teleoperation model: consists of different nodes to read out data from Kinect, Meta Quest, and Joycon Controllers and another module to control the robot. The blue lines indicate position data measured or actions on the operator side and resulting into motion or operation of the NAO robot. The red lines indicate visual data collected on the robot and provided as feedback to the operator.

The control structure of the software implementation and its architecture is depicted in Figure 5. The blue lines represent data provided by the operator to control the robot’s motion. In contrast, the red lines represent the data provided by the NAO to have a visual confirmation of the robot movements from a first-person perspective. As observed, the three devices had independent communication with the robot, allowing it to divide the teleoperation process in parallel actions that control each of them as part of the robot.

The control of the robot is made by the use of the controls to interact with a Human Machine Interface (HMI) to select one of the two teleoperation modalities, which can be seen in the Algorithm 1, which are explained in more detail in the next subsection.
**Algorithm 1:** Main menu of the teleoperation system1:**procedure**ROS NAO Node(Nodes and Topics)2:**Create** ROS NAO Node3:**Create** ROS Topic Subscriber to Kinect topic;4:**Create** ROS Topic Subscriber to Meta Quest topic;5:**Create** ROS Topic Subscriber to left Joycon topic;6:**Create** ROS Topic Subscriber to the right Joycon topic;7: **for** ROS NAO node exist **do**8:  **Print** “Welcome to the NAO teleoperation system”9:  **Print** “Press “START” to continue”10:  **if** Joycon “START” button is pressed **then**11:   **if** Joycon “X” button is pressed **then**12:    **Enter** Joycon teleoperation mode;13:    **if** Joycon “B” button is pressed **then**14:     **break**;15:    **end if**16:   **end if**17:  **end if**18:  **if** Joycon “A” button is pressed **then**19:   **Enter** Full teleoperation mode;20:   **if** Joycon “B” button is pressed **then**21:    **break**;22:   **end if**23:  **end if**24: **end if**25: **Destroy** ROS NAO Node;26: **Destroy** ROS Topic Subscriber to Kinect topic;27: **Destroy** ROS Topic Subscriber to Meta Quest topic;28: **Destroy** ROS Topic Subscriber to left Joycon topic;29: **Destroy** ROS Topic Subscriber to the right Joycon topic;30:**end procedure**

### 4.1. Joycon Teleoperation Mode

**Figure 6:**
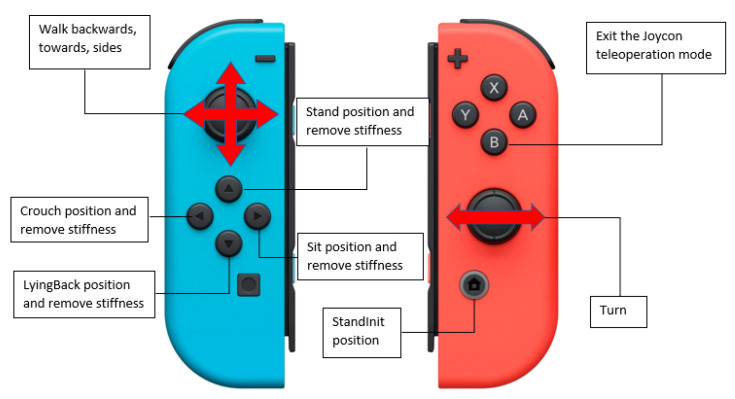
Button disposition of the Joycons.

The first mode of teleoperation aims to move the robot from one distance to another in the easiest way possible for the operator. In this mode, the user only has to manipulate the Nintendo Switch controllers [37] to move the robot forward, backward, to the sides, and even turn. Some buttons are also included to make the robot perform certain pre-programmed poses, such as sitting, lying down, or standing to be ready for a walking movement. This disposition can be seen in Figure 6. The movement begins by pressing the “START” button, which puts the robot in the initial position (“StandInit”) and enables the stiffness of the robot’s joints to control its mobility. Then the robot is ready to be moved by the thumbsticks in any direction. When button “B” is pressed, the operator exits this teleoperation and goes to the application’s main menu. This mode is also compatible with the Meta Quest use; the operator can move the robot’s head and receive the image of its camera without interfering with the NAO walking process.

### 4.2. Full Teleoperation Mode

**Figure 7:**
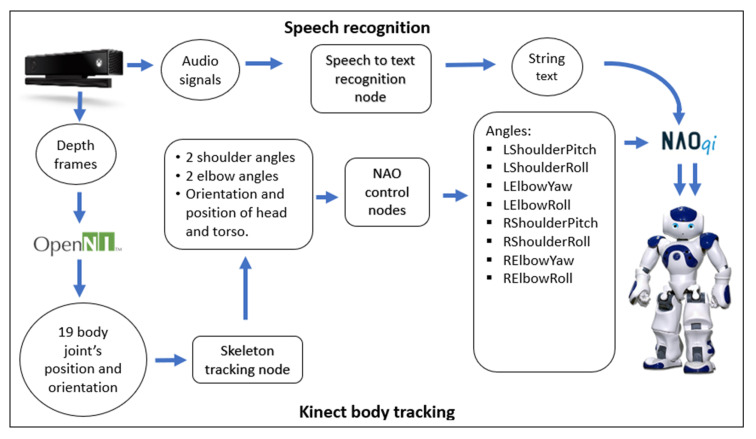
Software architecture of the data acquisition with the Kinect sensor.

In the second mode, three devices were used to control the NAO robot. The first is the Kinect sensor, which maps the user’s body skeleton joints, collecting information on the Cartesian position of the shoulder, elbow, and hand of the operator’s arms, and the position and orientation of the torso and head. The node treats this information of the device, where it transforms the position of the joints of each arm, in the angles that each joint of the arms of the NAO should move, as well as the position and orientation that the robot should have, with respect to its initial one. The information process to achieve the movement of the NAO robot with this device can be seen in more detail in Figure 7.

**Figure 8:**
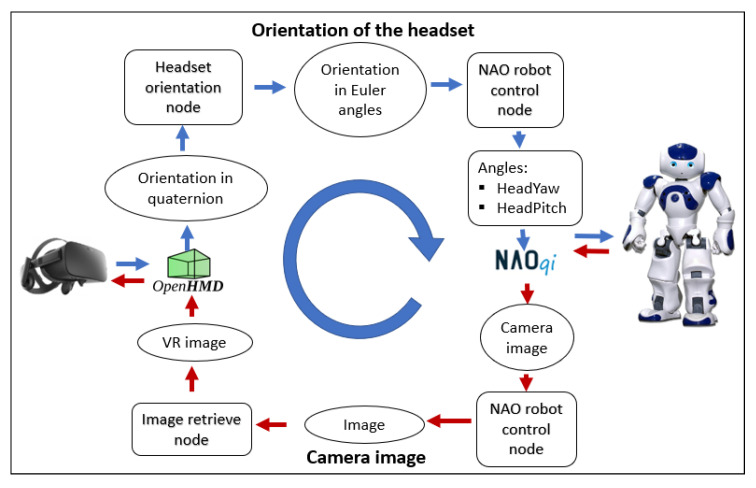
Software architecture of the data acquisition with the Meta Quest.

In the same way, the Meta Quest provides information from the headset orientation, which is collected in quaternions. This information is transformed into Euler’s angles (XYZ) and then treated to be sent to the robot as the angles of the head joints. At the same time, images of the robot’s front camera are modified to be used by the virtual reality glasses and send to them. The information process to achieve the movement of the robot’s head and the image retrieving can be seen in more detail in Figure 8.

**Figure 9:**
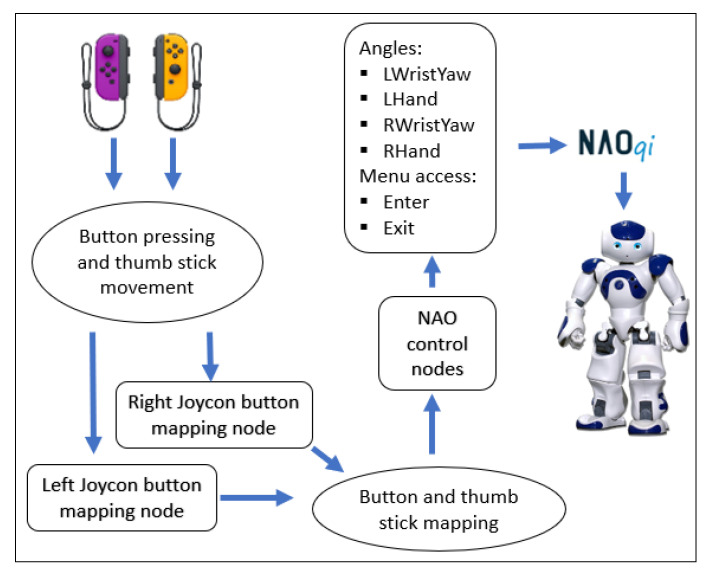
Software architecture of the data acquisition with the Joycon controllers.

**Figure 10:**
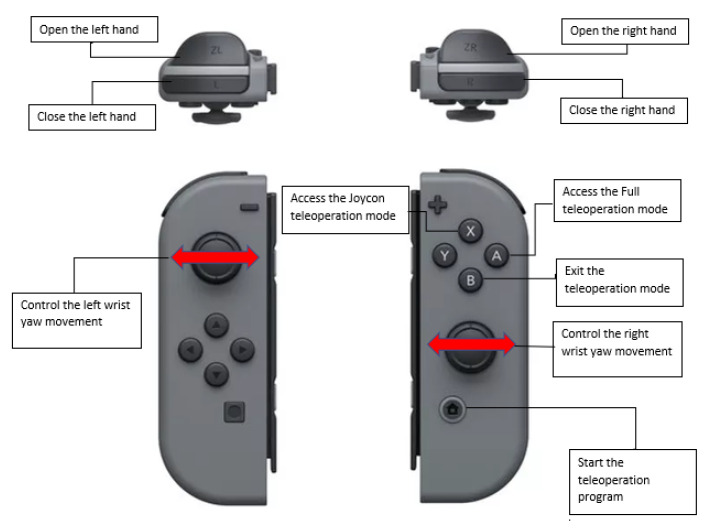
Button disposition of the Joycons.

Finally, the Nintendo Switch Joycons are used to control the wrist and hand joints by pressing certain buttons, as well as being able to enter and exit this teleoperation method. These controllers are implemented because the library used for tracking the human body does not recognize the movement of supination and pronation of the forearm and the fingers movements. Thus, to move the robots’ wrist joints, Joycon thumbsticks are used, and to open and close the hands, two buttons from each controller are used to perform each action. The arrangement of the buttons and thumbsticks in the controller for this modality is shown in Figure 9. The treatment process of the information received using the Joycons to control the robot is shown in Figure 10.

### 4.3. Node Architecture

**Figure 11:**
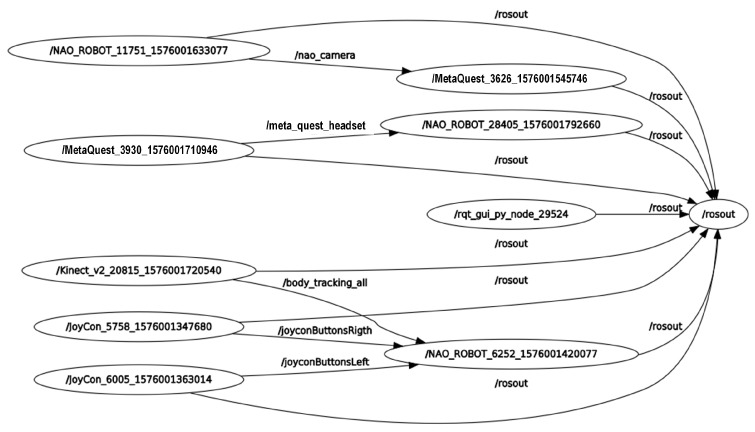
ROS rqt_graph, including the nodes and topics.

A publisher/subscriber communication is used so that a parallel programming architecture can handle the sensing devices and the NAO robot, managing several nodes to control them. This kind of programming improves the data transmission and allows independence in non-sequential procedures that govern the robot’s movements or functionality. Furthermore, message formats with predefined types of variables are used for universal purposes within the project. So, sent data can be read and used regardless of the programming language in which a node was developed. An ROS rqt_graph, including the nodes and topics, is shown in Figure 11.

### 4.4. Motion Capture

**Figure 12:**
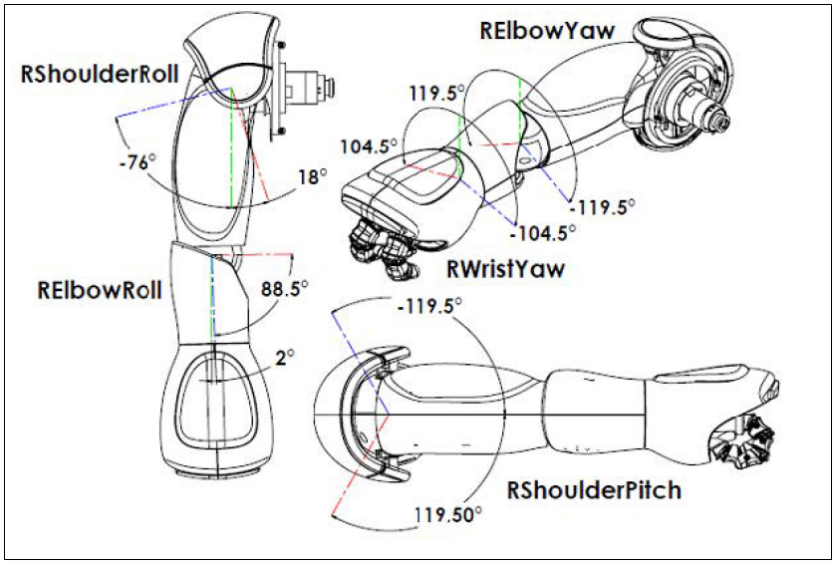
Right arm joint of the NAO robot [27].

As mentioned before, the libraries OpenNI2 and NiTE2 provide the position and orientation data of 15 joints in the human body. For the mapping of the operator’s arms, the shoulder, elbow, and hand positions for each arm were used. However, to make this mapping, it is necessary to clarify the correspondence of each joint of the arms of NAO to a corporal movement: the joint disposition of the right arm joint is shown in Figure 12. As for the shoulder, there are two degrees of freedom, the “ShoulderPitch” and “ShoulderRoll” joints. The first is equivalent to the movement of abduction and adduction of the shoulder, while the second corresponds to the shoulder flexion and extension movement. In the case of the elbow, there are also two degrees of freedom, “ElbowRoll” and “ElbowYaw”. The first is equivalent to flexion and extension of the elbow, whereas the second is equivalent to internal and external shoulder rotation. Finally, in the case of the wrist, there is the “WristYaw” joint, which is equivalent to the movement of pronation and supination of the elbow.

**Figure 13:**
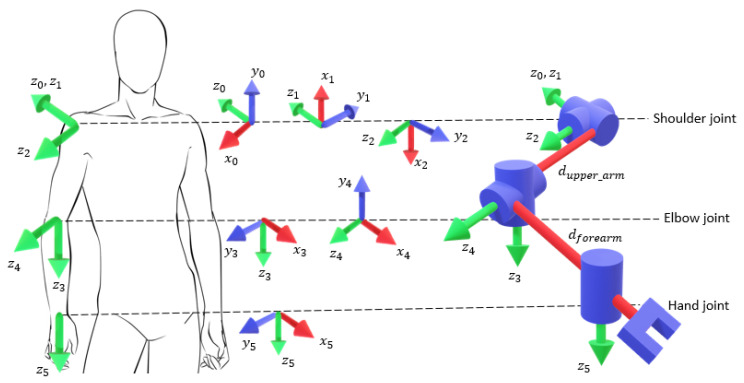
Link frame attachments to the human right limb.

With these concepts clear, an analytical approach to the reverse kinematics of each robot arm can be made, as explained in the work of Kofinas, Orfanoudakis, and Lagoudakis [38]. The representation of each of the arm manipulators of the robot can be represented as the plot of five joints according to the Modified Denavit–Hartenberg Parameter shown in Table 1 and Figure 13.

**Figure 14:**
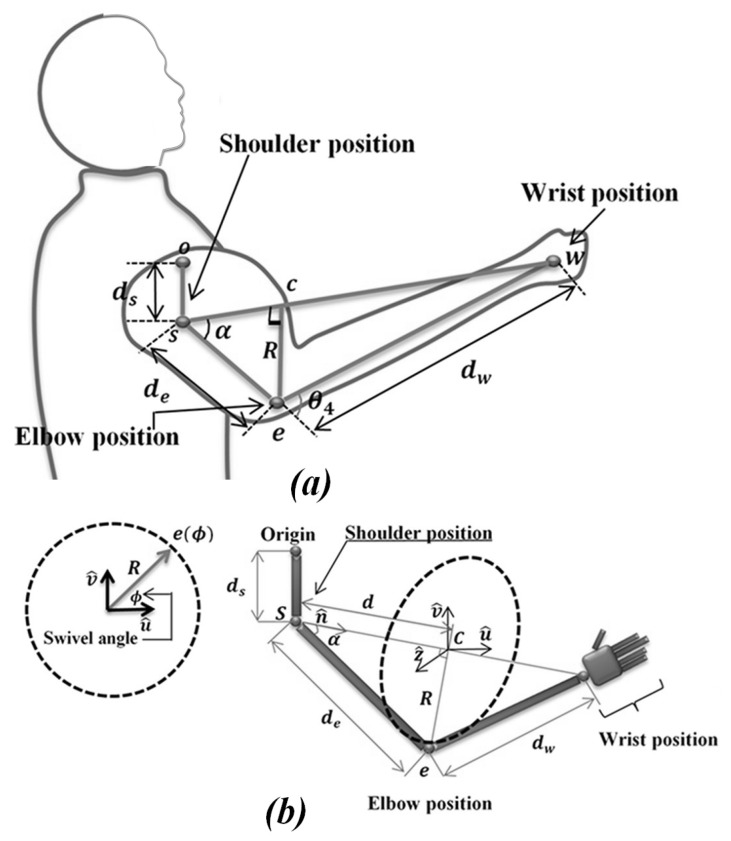
(**a**) Analysis of human upper limb motion. (**b**) Swivel angle (ϕ) configuration [39].

The upper limb configuration of humanoid robots, such as the NAO robot, is inspired by the human upper limb. The NAO robot has a redundant kinematics configuration that permits it to perform multiple motions as humans. However, there are infinite solutions to its inverse kinematics issue because of its redundancy structure. Usually, a human uses an optimal joint configuration of the arm when performing motion in space. The elbow position is determined via the first three (shoulder) joints, while its angle (elbow angle) is defined from the wrist position. The rotation configuration of the wrist is determined by the last three joints of the wrist portion. Based on the above analysis of the human upper limb motion, the inverse kinematic solution of NAO’s upper limb is inspired. The elbow angle $\theta_{4}$ (see Figure 14a) is defined geometrically from the known final destination, the end-effector of the NAO’s upper limb as:

(1) $$\theta_{4}=\pi \pm acos\left(\frac{d_{w}^{2}+d_{e}^{2}-{∥w-s∥}^{2}}{2d_{e}d_{w}}\right)$$

The distance (*R*) and angle (α) situated between $d_{e}$ and the virtual axis that links the end-effector position and the shoulder position permit us to determine the elbow position. From this position, it is easy to determine $\theta_{1}$, $\theta_{2}$, $\theta_{3}$ analytically. From the known rotation of the end-effector and angles ($\theta_{1}$, $\theta_{2}$, $\theta_{3}$, and $\theta_{4}$), the joint $\theta_{5}$ can be defined easily by the comparison, as given in Section 4.4.3.

When positioning the NAO in special poses, the elbow can be considered to be unrestricted in its rotation about an axis determined by swivel angle (ϕ) to the shoulder. The axes of the circle point at the shoulder joint and the wrist joint, meaning that they are perpendicular to the direction of motion. Swivel angle (ϕ) is the virtual angle between an elbow’s rotation relative to a virtual axis attached to the shoulder and wrist joints. As the swivel angle changes, the elbow outlines the arc of a circle on a plane whose normal is parallel to the shoulder-to-wrist axis, as shown in Figure 14b. In general, a simple motion does not impose any restrictions on the wrist position, allowing the wrist to be fixed at the desired swivel angle. However, this fixation has no effect on the redundancy of the NAO’s arm. To illustrate the circle from a mathematical perspective, the normal vector of the plane can be defined as follows:

(2) $$\hat{n}=\frac{w-s}{∥w-s∥}$$

Then, $\hat{u}$ is set to be a projection of an axis $\hat{z}$ selected arbitrarily on the circle:

(3) $$\hat{z}={\left[0\;\;\;0\;\;\;1\right]}^{T}$$

(4) $$\hat{u}=\frac{\hat{z}-\left(\hat{z}\;\hat{n}\right)\hat{n}}{∥\hat{z}-\left(\hat{z}\;\hat{n}\right)\hat{n}∥}$$

and $\hat{\upsilon }$ is the last component of the orthonormal base:

(5) $$\hat{\upsilon }=\hat{n}\times \hat{u}$$

*R* and *c* are the radius and center of the circle, respectively. The radius *R* is obtained with simple trigonometric relationships. By defining the distance *d* or center of circle *c* as (see Figure 14b):

(6) $$d=Cos\left(\alpha \right)d_{e}\hat{n}$$

with:

(7) $$Cos\left(\alpha \right)=\frac{d_{w}^{2}-d_{e}^{2}-{∥w-s∥}^{2}}{-2d_{e}∥w-s∥}$$

and:

(8) $$R=Sin\left(\alpha \right)d_{e}$$

Finally, the elbow position can be characterized as a function of the swivel angle (ϕ) about axis $\hat{u}$:

(9) $$e\left(\phi \right)=d+R\left[Cos\left(\phi \right)\hat{u}+Sin\left(\phi \right)\hat{\upsilon }\right]+s$$

where *s* is the vector shoulder coordinate determined as $s_{shoulder}={\left[0\;\;0\;\;d_{s}\right]}^{T}$; (see Equation (10)). The shoulder is translated by a distance $d_{s}$ on the z-axis as regard to the origin (frame $\left\{0\right\}$ in Figure 12).

(10) $$P_{shoulder}{=}_{1}^{0}T{\left[0\;\;0\;\;0\;\;1\right]}^{T}={\left[0\;\;0\;\;d_{s}\;\;1\right]}^{T}$$

where ${}_{1}^{0}T$ is the first homogeneous transformation matrix obtained by using Table 1. The elbow position is obtained only via the three joints of the shoulder portion. $\theta_{1}$, $\theta_{2}$ and $\theta_{3}$:

(11) $$P_{elbow}{=}_{1}^{0}T{\;}_{2}^{1}T{\;}_{3}^{2}T{\left[0\;\;0\;\;0\;\;1\right]}^{T}={\left[e_{x}\;\;e_{y}\;\;e_{z}\;\;1\right]}^{T}$$

so,

(12) $$P_{elbow}=\left[\begin{array}{c} e_{x}\\ e_{y}\\ e_{z} \end{array}\right]=\left[\begin{array}{c} d_{e}C_{1}S_{2}\\ d_{e}S_{1}S_{2}\\ d_{s}+d_{e}C_{2} \end{array}\right]$$

Here, $C_{i}$ denotes $Cos(\theta_{i})$, and $S_{i}$ denotes $Sin(\theta_{i})$. The wrist joint position depends on $\theta_{1}$, $\theta_{2}$, $\theta_{3}$, and $\theta_{4}$. The last joint $\theta_{5}$ is used to define the wrist orientation.

(13) $$\begin{array}{cc} P_{elbow} & {=}_{1}^{0}T{\;}_{2}^{1}T{\;}_{3}^{2}T{\;}_{4}^{3}T{\;}_{5}^{4}T{\left[0\;\;0\;\;0\;\;1\right]}^{T}={\left[w_{x}\;\;w_{y}\;\;w_{z}\;\;1\right]}^{T}\\ \; & =\left[\begin{array}{c} d_{e}C_{1}S_{2}-d_{w}\left(S_{4}\left(S_{1}S_{3}-C_{1}\right)C_{2}C_{3}\right)-C_{1}C_{4}S_{2}\\ d_{w}\left(S_{4}\left(C_{1}S_{3}+C_{2}C_{3}S_{1}\right)+C_{4}S_{1}S_{2}\right)+d_{e}S_{1}S_{2}\\ d_{s}+d_{w}\left(C_{2}C_{4}-C_{3}S_{2}S_{4}\right)+d_{e}C_{2} \end{array}\right] \end{array}$$

#### 4.4.1. Solving $\theta_{1}$ and $\theta_{2}$

Obtained from Equation (12), the elbow position is known. So:

(14) $$\begin{array}{c} \frac{e_{y}}{e_{x}}=\frac{d_{e}S_{1}S_{2}}{d_{e}C_{1}S_{2}}\Rightarrow \theta_{1}=atan2\left(e_{y},e_{x}\right) \end{array}$$

$\theta_{2}$ has two solutions, i.e., an analytic solution and a geometric solution, from Equation (12):

(15) $$\begin{array}{c} Cos\left(\theta_{2}\right)=\frac{e_{z}-d_{s}}{d_{e}} \end{array}$$

Yields:

(16) $$\left\{\begin{array}{c} Sin\left(\theta_{2}\right)=\frac{e_{x}}{d_{e}C_{1}}\;\;\;if\;\;C_{1}\neq 0\\ Sin\left(\theta_{2}\right)=\frac{e_{y}}{d_{e}S_{1}}\;\;\;Otherwise \end{array}\right.$$

So,

(17) $$\theta_{2}=atan2\left(Sin\left(\theta_{2}\right),\frac{e_{z}-d_{s}}{d_{e}}\right)$$

**Figure 15:**
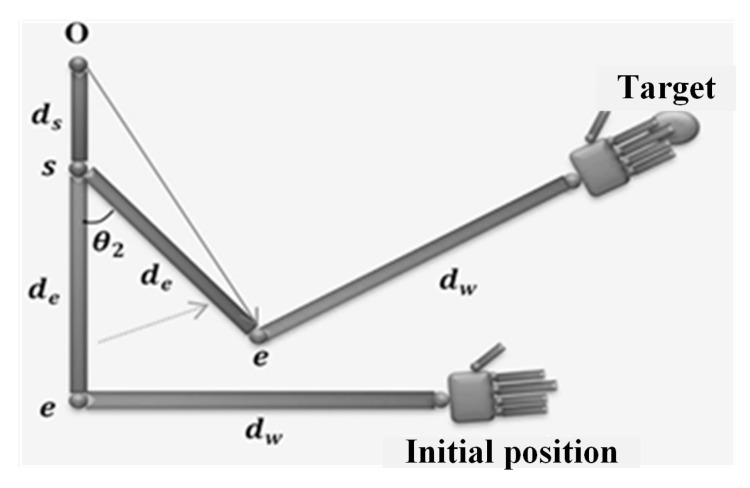
Joint $\theta_{2}$ geometrical solution [39].

Since joint 2 exists at the shoulder in Figure 15, the coordinate of the origin (joint 1 in Figure 14) is [000], the elbow position is determined by Equation (12), and the distance between the shoulder and elbow $d_{e}$ is constant. The joint $\theta_{2}$ can be obtained by using the law of cosines:

(18) $$\theta_{2}^{*}=\pi \pm acos\left(\frac{{∥e-O∥}^{2}-d_{e}^{2}-d_{s}^{2}}{-2d_{e}d_{s}}\right)$$

where $\theta_{2}^{*}$ is the geometric solution.

#### 4.4.2. Solving $\theta_{3}$

Joint 3 ($\theta_{3}$) has two solutions as well, analytic and geometric. To determine $\theta_{3}$ analytically, we multiply both sides of Equation (13) by ${\left({}_{1}^{0}T{\;}_{2}^{1}T\right)}^{-1}$:

(19) $$\begin{array}{c} {\left({}_{1}^{0}T{\;}_{2}^{1}T\right)}^{-1}\left({}_{1}^{0}T{\;}_{2}^{1}T{\;}_{3}^{2}T{\;}_{4}^{3}T{\;}_{5}^{4}T\right){\left[0\;\;0\;\;0\;\;1\right]}^{T}={\left({}_{1}^{0}T{\;}_{2}^{1}T\right)}^{-1}{\left[w_{x}\;\;w_{y}\;\;w_{z}\;\;1\right]}^{T} \end{array}$$

Yields:

(20) $$\begin{array}{c} \left[\begin{array}{c} d_{w}S_{4}C_{3}\\ -d_{w}C_{4}-d_{e}\\ d_{w}S_{4}S_{3}\\ 1 \end{array}\right]=\left[\begin{array}{c} d_{s}S_{2}-w_{z}S_{2}+w_{x}C_{1}C_{2}+w_{y}C_{2}S_{1}\\ w_{x}C_{1}S_{2}-w_{y}S_{1}S_{2}+d_{s}C_{2}-w_{z}C_{2}\\ w_{y}C_{1}-w_{x}S_{1}\\ 1 \end{array}\right]\\ \theta_{3}=atan2\left(w_{y}C_{1}-w_{x}S_{1},\;d_{s}S_{2}-w_{z}S_{2}+w_{x}C_{1}C_{2}+w_{y}C_{2}S_{1}\right) \end{array}$$

**Figure 16:**
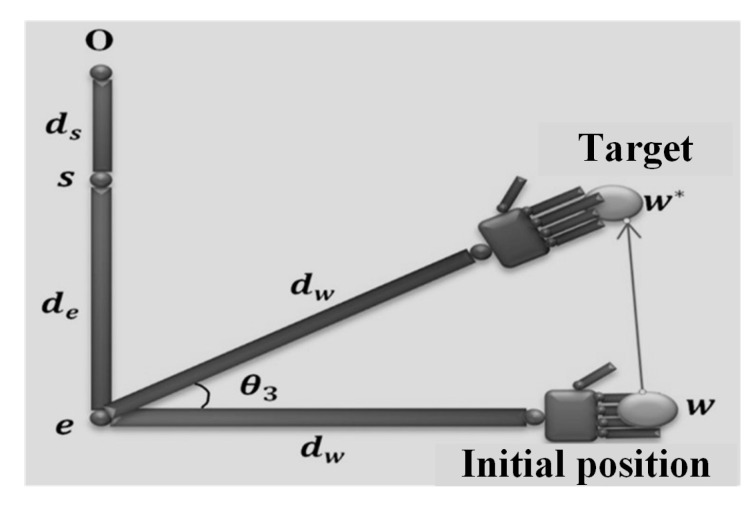
Geometrical solution of joint $\theta_{3}$ [39].

Using the law of cosines (see Figure 16, the geometric solution can obtained as:

(21) $$\theta_{3}^{*}=\pi \pm acos\left(\frac{∥w-w^{*}{∥}^{2}-2d_{w}^{2}}{-2d_{w}^{2}}\right)$$

where $\theta_{3}^{*}$ is the geometric solution.

Two solutions (analytic and geometric) have been obtained for each joint (joints 2 and 3). These solutions are valid for NAO’s upper limb movements.

#### 4.4.3. Solving $\theta_{5}$

The orientation of the end-effector is given by:

(22) $$\begin{array}{c} R_{end-effector}=R_{1}R_{2}R_{3}R_{4}R_{5}=\left[\begin{array}{ccc} r_{11} & r_{12} & r_{13}\\ r_{21} & r_{22} & r_{23}\\ r_{31} & r_{32} & r_{33} \end{array}\right] \end{array}$$

Consider that $R_{w}=R_{5}$. Substituting it in Equation (22):

(23) $$\begin{array}{c} R_{w}=R_{4}^{T}R_{3}^{T}R_{2}^{T}R_{1}^{T}R_{end-effector} \end{array}$$

where the $R_{end-effector}$ matrix defines the desired end-effector orientation with respect to the origin. In this condition, Euler angles are used and by comparison, wrist angles can be found as:

(24) $$\begin{array}{c} tan\left(\theta_{5}\right)=\frac{r_{33}}{r_{13}}⟹\theta_{5}=atan2\left(r_{31},\;r_{11}\right) \end{array}$$

**Figure 17:**
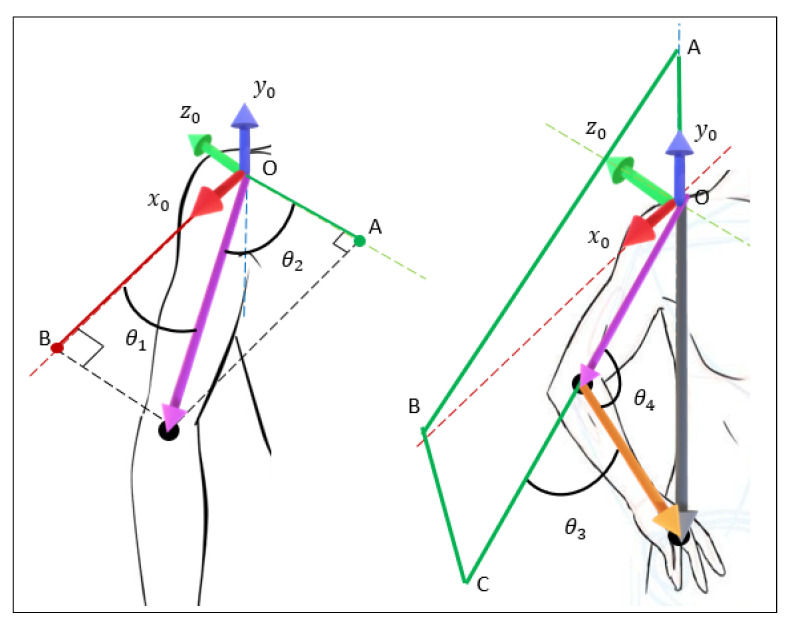
Arm angles calculation.

However, based on the work of Md Assad-Uz-Zaman [5], a geometric approach is made to simplify calculations and avoid points of the singularity of the manipulator when converting the three Cartesian positions of the arm (shoulder, elbow, and hand) into the orientation of the angles of the four joints of the arm (two from the shoulder and two from the elbow). First, to make the kinematic analysis of each arm, it is necessary to change the reference frame from the Kinect to the shoulder, which will have the same orientation, transforming the joint position as the subtraction of the Cartesian coordinates between the shoulder and the joint. So, the shoulder coordinates are (0,0,0), and the elbow and hand coordinates will be noted as ($x_{e}$,$y_{e}$,$z_{e}$) and ($x_{h}$,$y_{h}$,$z_{h}$), respectively. The distance between the shoulder and the elbow is noted as $d_{upper\_arm}$, the distance between the elbow and the hand as $d_{forearm}$, and the distance between the shoulder and the hand as $d_{arm}$. With this, a vector OA is considered as the projection of the vector $d_{upper\_arm}$ (from the origin to the elbow position) on the z-axis, being the coordinates of A at (0, 0,−$z_{elbow}$). Now, the angle between $d_{upper\_arm}$ vectors OA is the angle $\theta_{1}$. Further, consider a vector OB as the projection of the vector $d_{upper\_arm}$ on the x-axis, locating point B at ($x_{elbow}$,0,0) position. Therefore, the angle between OB and $d_{upper\_arm}$ is the angle $\theta_{2}$. Angle calculations are shown in Figure 17.

Then,

(25) $$cos\theta_{1}=\frac{-z_{e}}{\sqrt{x_{e}^{2}+y_{e}^{2}+z_{e}^{2}}}$$

Furthermore,

(26) $$\theta_{1}=arccos\left(cos\theta_{1}\right)$$

In a similar way,

(27) $$cos\theta_{2}=\frac{x_{e}}{\sqrt{x_{e}^{2}+y_{e}^{2}+z_{e}^{2}}}$$

Therefore, “Equation (25)” is used to obtain $\theta_{2}$. Where, if $y_{e}<0$, $\theta_{2}=-\theta_{2}$. For angle $\theta_{3}$, consider a plane OABC that passes through $y_{0}$ axis and upper arm vector. Then, the angle between this plan and forearm is $\theta_{3}$.

(28) $$sin\theta_{3}=\frac{z_{e}\left(x_{h}-x_{e}\right)-x_{e}\left(z_{h}-z_{e}\right)}{\sqrt{x_{e}^{2}+y_{e}^{2}+z_{e}^{2}}\sqrt{{\left(x_{h}-x_{e}\right)}^{2}+{\left(y_{h}-y_{e}\right)}^{2}+{\left(z_{h}-z_{e}\right)}^{2}}}$$

Additionally,

(29) $$cos\theta_{3}=\pm \sqrt{1-{\left(sin\theta_{3}\right)}^{2}}$$

Therefore,

(30) $$\theta_{3}=arctan\left(\frac{\pm sin\theta_{3}}{cos\theta_{3}}\right)$$

Finally, the joint angle $\theta_{4}$ is determined geometrically using the cosine rule.

(31) $$\begin{array}{cc} {\left(d_{arm}\right)}^{2}={\left(d_{upper\_arm}\right)}^{2}+{\left(d_{forearm}\right)}^{2} & \\ -2d_{upper\_arm}d_{forearm}cos\theta_{4} \end{array}$$

Then,

(32) $$\theta_{4}=arccos\left(\frac{{\left(d_{upper\_arm}\right)}^{2}+{\left(d_{forearm}\right)}^{2}-{\left(d_{arm}\right)}^{2}}{2d_{upper\_arm}d_{forearm}}\right)$$

To match the joint angles of the human body with the NAO robot joint orientation, it is necessary to introduce some small changes in the calculation. The modified angles are listed in Table 2.

**Figure 18:**
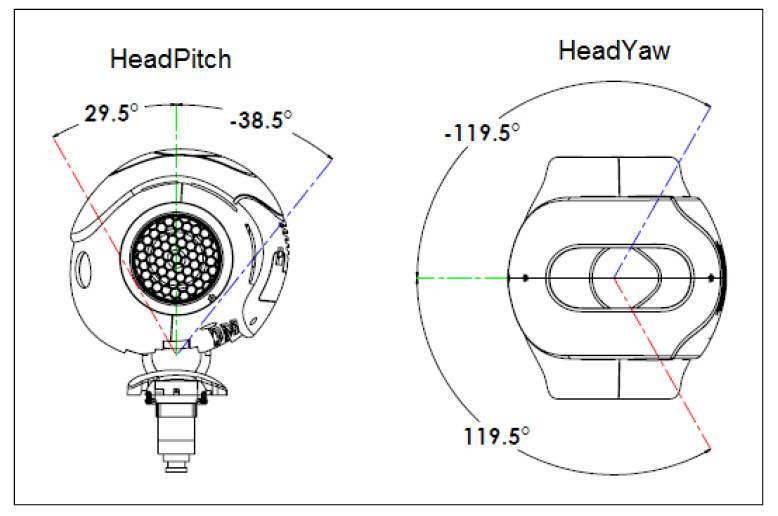
Head joint of the NAO robot [27].

Another motion tracking that should be taken into account is the NAO’s head, controlled by the headset. It has two joints, the “HeadPitch”, which is equivalent to flexion and extension of the neck, and the “HeadYaw”, which is the lateral rotation to the left and right (Figure 18). The quaternions retrieve from the gyroscope of the headset are transformed into Euler’s angles (XYZ), which have to be the equivalent of the robot joints, as shown in Table 3.

### 4.5. Image Retrieving

**Figure 19:**
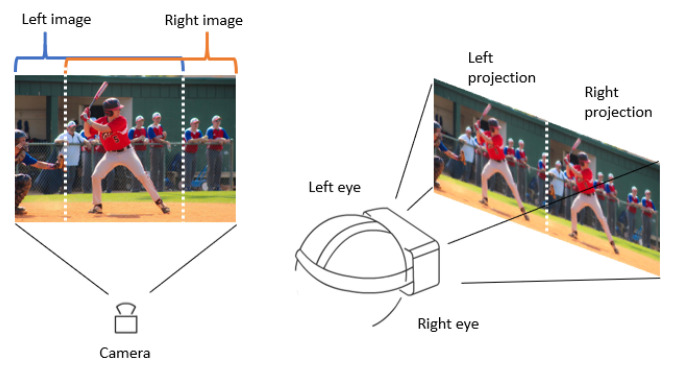
Treatment of the camera image.

To transmit images directly into the Meta Quest, several conditions need to be considered. Normally, the separation of the human eyes is on average 6 cm [40], which makes each of the eyes see the world from a scarcely different perspective. The brain joins these two views to create a sense of depth. However, if the camera image is sent directly to the headset, having a display that is not divided by eye, and due to the screen’s proximity in sight, the operator cannot comfortably see what the camera is receiving. That is why a quick approach to an immersive virtual reality experience can be made through an image treatment, where the Meta screen should be split into two, having an independent image for each eye. This is done to show 80 percent of the image from the left side and 80 percent from the right side to create the illusion of a stereoscopic image. The process is shown in Figure 19. In this way, a first-person perspective of the robot is easily created. The control of the joints of the robot’s head by the headset orientation builds an immersive experience of the teleoperation.

## 5. Results and Discussion

### 5.1. Head Motion Mimic

**Figure 20:**
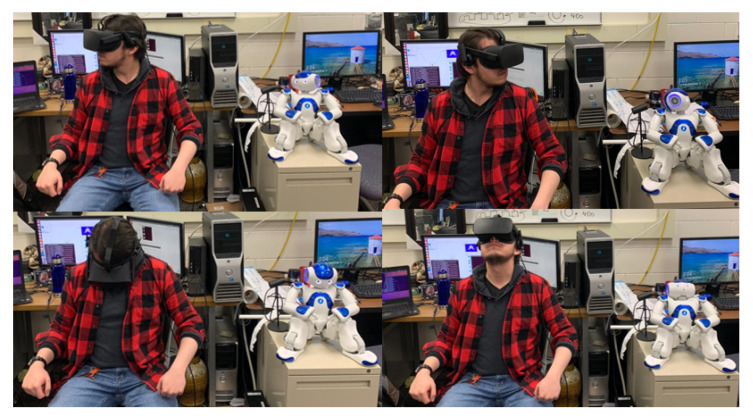
Process of imitation for NAO robot head following.

**Figure 21:**
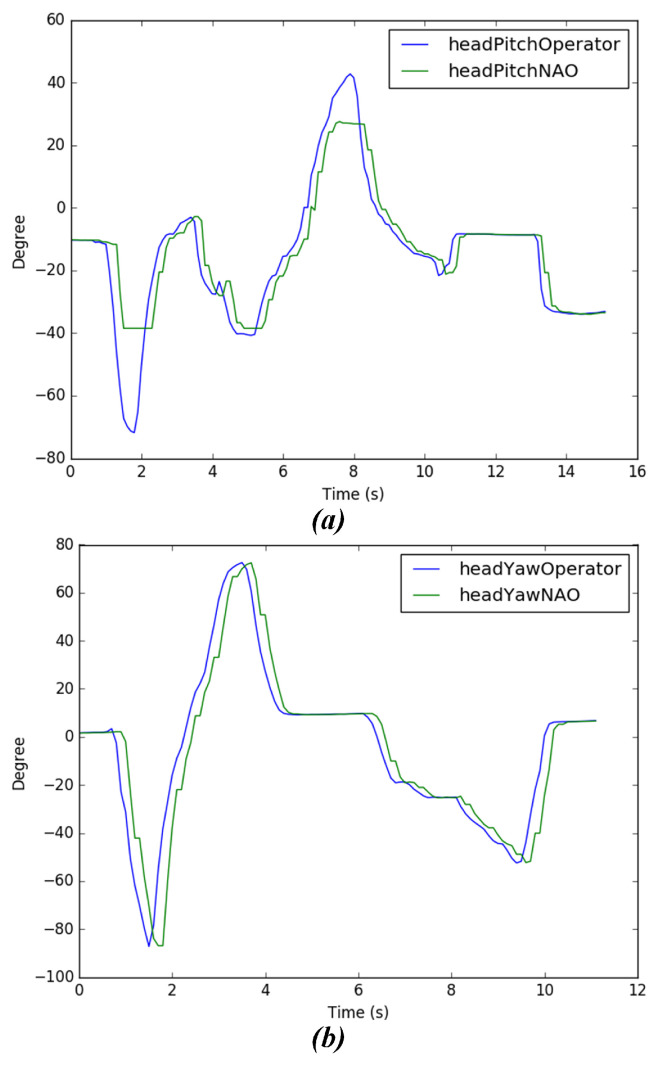
Result of head following experiment for both human and NAO robot. (**a**) Head pitch motion (**b**) Head Yaw motion.

In this part, we experiment to test the stability of the head following system, in which a person puts the Meta Quest headset on and moves their head to move the NAO’s head. First, the operator will rotate their head 80° to the left direction; then they will rotate 80° to the right direction, pitching up with 40° and pitching down with 70°, respectively. Figure 20 shows the process of the experiment. Figure 21a,b graphs were created, wherein data has been retrieved from head movement angles from the operator and the robot. From the result of the head following experiment, the movement of the user’s head can be followed accurately, allowing the operator to have a first-person view from the camera of the robot. However, it can be seen, especially in Figure 21a that the joints of the robot do not reach angles that the operator does due to design restrictions. However, it can be observed that the joints of the robot in both the yaw and pitch movements do not reach angles that the operator achieves, as per design restrictions [41,42]. Nevertheless, if the operator’s head movement exceeds the limits of movement of the robot’s joints, it will move to the closest angle it can.

### 5.2. Arm Motion Mimic

**Figure 22:**
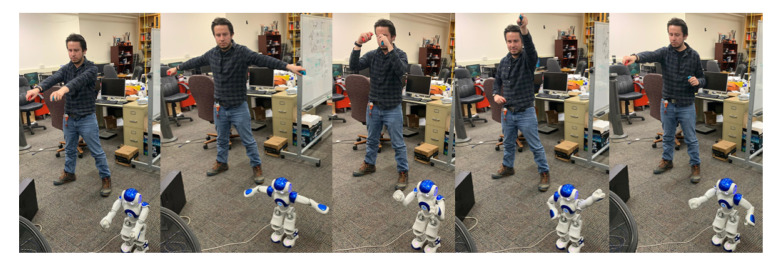
Process of imitation for NAO robot upper limb following.

**Figure 23:**
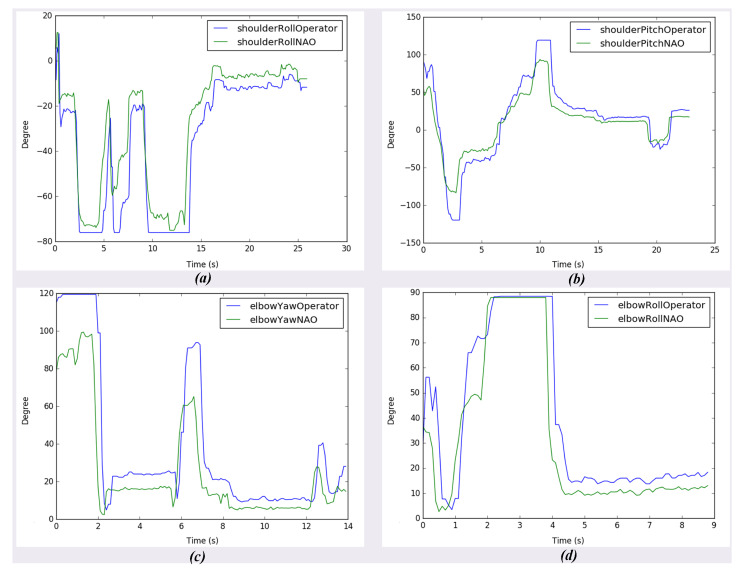
Result of upper limb following experiment for both human and NAO robot. (**a**) Shoulder roll motion (**b**) Shoulder pitch motion (**c**) Elbow yaw motion, and (**d**) Elbow roll motion.

In the second experiment, the operator moves his upper limbs to the Kinect camera to retrieve his movements. Again, the operator carried out different positions shown in Figure 22 to evaluate how precise and accurate the robot’s movements are. The operator is asked to make arm movements that only move one of the joints that define the degrees of freedom of the robot arms to collect the information read by the Kinect in comparison to that read by the sensors of the robot. These joints are ShoulderRoll, ShoulderPitch, ELbowYaw, and ELbowRoll. As a result, a series of graphs can be seen in Figure 23, such as the mapping of the human body, when the arms are facing the chest, which causes the sensor to make mistakes when interpreting the movement joints of the operator, making the robot’s movement not precise. In addition, the fact that for effective body tracking, the operator must be as the front of the sensor as possible, because if he rotates, the interpretation of the movement of the arms becomes inaccurate, by mapping the operator’s torso incorrectly and at the same time the position of the furthest arm from the camera. However, when the operator stays in front of the sensor, the robot follows the operator’s movement in the four joints that are mapped with this sensor continuously.

### 5.3. Body Motion Mimic

**Figure 24:**
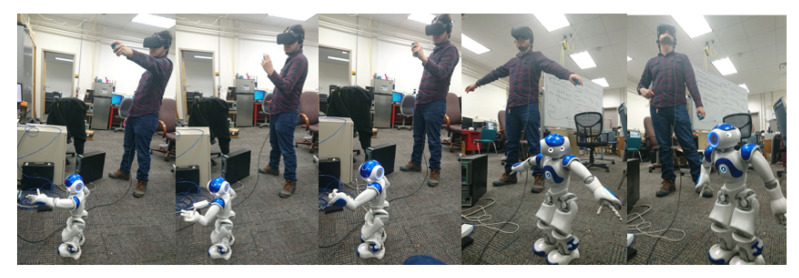
Process of imitation for NAO robot joint following.

In the following experiment, the entire Kinect, Meta Quest, and Joycons are used. We want to evaluate the use of the three devices simultaneously to control the robot’s movement to see if there is any communication problem when the three devices are in action. In addition, the change in teleoperation modality is also evaluated through the controls to see the independence of the operator in the process, as can be seen in Figure 24.

**Figure 25:**
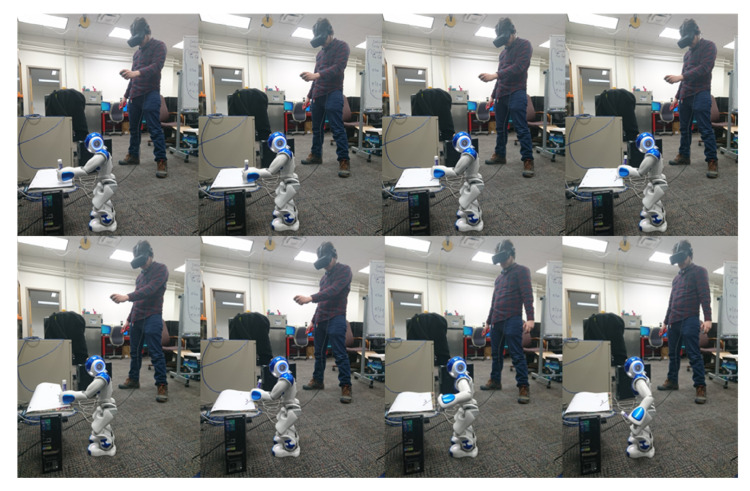
Result of marker manipulation with the NAO robot.

It was observed that the use of ROS allows the control of different sections of the robot without any problem in the loss of information because of default TCPROS transport layer of messages and services. In multi-modal teleportation where UDP transport protocol is used, a loss of information is observed [43]. In addition, it was observed that the change between teleoperation modalities is done quickly by utilizing the buttons of the Joycons, which allows this developed process to have the facility of only needing the operator to control the entire process. One aspect to be highlighted is that the first perspective vision with the NAO camera is not as immersive as expected, making the movements of the arms from the head’s perspective feel not as natural as the movements of the operators’ arms seen from his perspective. Nonetheless, another experiment in which the NAO held a market in its hand will be teleoperated to draw lines on a paper. As shown in Figure 25, the operator can control the robot’s hand and see what the robot is doing with the headset. Nevertheless, the teleoperation experience can be improved by controlling the torso orientation to limb the robot and had more workspace than just the reach area of both hands when the robot is standing on its foot.

### 5.4. Safety Routines

There have been plenty of safety routines when the teleoperation protocol is operative. When using the Kinect, the user must be in full view of the sensor. The entire body is adequately swallowed, and the movement is not misinterpreted by being hidden or obscured by some other object. Therefore, two requirements must be ensured before beginning teleoperation. The first one is that the sensor does not find more than one person in the visibility zone of the Kinect since having two or more people could result in the interpretation of a human skeleton with the mobile joints of all people within the visibility area. The second one is that when obtaining the information of the 19 traceable joints of the human body by the sensor, they have to have a confidence level equal to one to guarantee the least amount of noise between the recovered data. The teleoperation will stop if one of these requirements is not fulfilled. It is also worth highlighting the use of Nintendo Switch controls. Because of their ergonomics, they can allow the separation of both arms, compared to any other video game control. Still, these enable the operator to interact with the teleoperation software without needing a second person to configure and implement the protocol. Furthermore, during the experimental phase, it was found that validating the teleoperation and safety routines in a safe environment is always important. For this purpose, the Choreographer simulated the version on the NAO connected via Naoqi to create the first tests. Nonetheless, it is essential to acknowledge that the NAO robot acts more stable in the simulated world than in the real world.

### 5.5. Discussion

**Figure 26:**
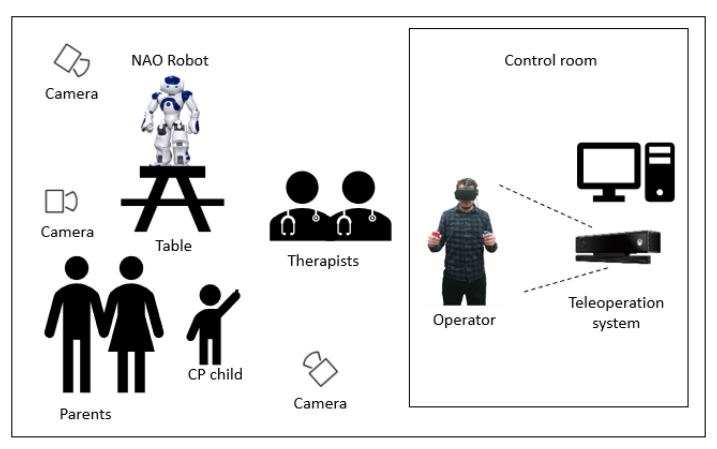
Draft of a room setup for the experiment.

The proposed approach was validated using the Kinect V2 sensor, Meta Quest headset, Nintendo Switch Joycons, and the NAO robot. There is ease between changing the operating modes of the robot, using only the controls, which allows the robot to move in different points easily. More importantly, it allows the follow-up of the arm movements as the operator’s head easily, allowing him to have an immersive experience when controlling the robot, seeing it from the robot’s point of view and speaking through it. Sources of latency were considered originating from the hardware and physical limitations of communication. While transferring videos and motion data we experienced negligible latency (<100 ms). It is clear to think that there are different applications for this telepresence, such as long-distance teaching with active users’ active participation. Moreover, taking into account the work of it, it is possible to say that there is potential to carry out interaction therapies with autistic children [44,45,46,47,48], improving the communication skills of the patients. Therefore, based on the work of Malik, Yussof, and Hanapiah in [49] and Shamsuddin et al. in [50], a therapy work could be proposed with the following experimental layout to analyze the advantages of the teleoperation system to be used for therapeutic purposes, as shown in Figure 26.

However, regarding future work, there are many possibilities to improve the teleoperation system. For example, the controls of Nintendo Switch are optimal for the proposed teleoperation, being ergonomic, and allowing the separation of the operator’s arms when the manipulation of the robot is in progress. Unfortunately, a driver is not found at the moment that allows the IMU of the Joycons to be collected in Linux. Therefore, one of the first tasks to improve this system is to access their information to control the movement of the robot’s wrist and control their HD rumble with haptic teleoperation purposes by receiving feedback from the environment in which the robot’s hands are interacting. At the same time, using a real-sense camera instead of one that the robot has in its forehead will allow a more immersive experience having more resolution and panoramic vision than the one currently used. This would allow the mapping of the place in real-time by the depth camera functionality, potentially using the robot’s trajectories. However, to use this type of camera, it is required that the robot carries a raspberry pi with it so that the video transmission of the camera does not depend on cables, restricting the movements of the NAO. Finally, creating an application in a virtual reality environment that allows more control of the teleoperation from the VR glasses and not from the computer screen display is advised.

## 6. Conclusions

From the experimental results, it can be concluded that the teleoperation framework developed in this study can be used for therapeutic purposes. Our designed control architecture and its integration with other components in ROS platform make this teleoperation possible. The proposed approach is validated through the robot’s movement to perform different assignments. The gesture teleoperation system, together with the user interface, allows the robot to imitate arm movements and act according to the operator’s predefined body movements simultaneously. The proposed approach offers an architecture that can have many applications beyond pure teleoperation within the area of socially assistive robotics, where robots assist through social interaction. Many authors agree that humanoid robots can help assist patients with disabilities or pediatric therapy such as physiotherapy because children tend to accept robots more naturally than adults, and they are motivated to replicate robot’s movement as a therapy. The developed software could help the therapists without programming skills record the exercises on the robot and provide a tool for recording or learning sequences of poses from the mimicked movements.

## Figures and Tables

**Table 1:** Modified Denavit–Hartenberg parameters.

Joint(i)	$a_{i-1}$	$d_{i}$	$\alpha_{i-1}$	$\theta_{i}$
1	0	0	0	$\theta_{1}$−π/2
2	π/2	0	0	$\theta_{2}$
3	π/2	$d_{upperarm}$	0	$\theta_{3}$
4	−π/2	0	0	$\theta_{4}$
5	π/2	$d_{forearm}$	0	$\theta_{5}$

**Table 2:** NAO joint angles.

Angle	NAO Joint	Modified Angle
$\theta_{1}$	RShoulderRoll	$\theta_{1}$− 90°
$\theta_{2}$	RShoulderPitch	$\theta_{2}$
$\theta_{3}$	RElbowYaw	90°−$\theta_{3}$
$\theta_{4}$	RElbowRoll	180°−$\theta_{4}$
$\theta_{1}$	LShoulderRoll	90°−$\theta_{1}$
$\theta_{2}$	LShoulderPitch	$\theta_{2}$
$\theta_{3}$	LElbowYaw	−90°−$\theta_{3}$
$\theta_{4}$	LElbowRoll	$\theta_{4}$−180°

**Table 3:** NAO head angles.

Angle	OR Angle Name	NAO Joint	Modified Angle
$\theta_{1}$	Roll	HeadPitch	$\theta_{1}$
$\theta_{2}$	Pitch	HeadYaw	$-\theta_{2}$

## Data Availability

The data collected and analyzed during this study are available from the corresponding author upon reasonable request.
